# Staring at the Cold Sun: Blue Light Regulation Is Distributed within the Genus *Acinetobacter*

**DOI:** 10.1371/journal.pone.0055059

**Published:** 2013-01-24

**Authors:** Adrián Golic, Mario Vaneechoutte, Alexandr Nemec, Alejandro M. Viale, Luis A. Actis, María Alejandra Mussi

**Affiliations:** 1 Microbiology Division, Instituto de Biología Molecular y Celular de Rosario (IBR- CONICET), Facultad de Ciencias Bioquímicas y Farmacéuticas, Universidad Nacional de Rosario, Rosario, Santa Fe, Argentina; 2 Department of Microbiology, Miami University, Oxford, Ohio, United States of America; 3 Laboratory for Bacteriology Research, Ghent University Hospital, Ghent University, Ghent, Belgium; 4 Laboratory of Bacterial Genetics, National Institute of Public Health, Prague, Czech Republic; Loyola University Medical Center, United States of America

## Abstract

We previously showed that the opportunistic nosocomial pathogen *Acinetobacter baumannii* is able to sense and respond to light via BlsA, a BLUF (Blue-Light-sensing Using FAD)-domain photoreceptor protein. Here, we extend our previous studies showing that light regulation is not restricted to *A. baumannii*, but rather widespread within the genus *Acinetobacter*. First, we found that blue light modulates motility and biofilm formation in many species of the genus, including members of the *Acinetobacter calcoaceticus-A. baumannii* complex. In many of these species blue light acts as a key factor guiding the decision between motility or sessility at 24°C, whereas in *A. baumannii*, light inhibits both motility and biofilm formation. We also show that light regulation of motility occurred not only at 24°C but also at 37°C in non-*A. baumannii* species, contrasting the situation of *A. baumannii* which only shows photoregulation at 24°C. Second, we show that *Acinetobacter baylyi* (strain ADP1) BLUF-photoreceptors can functionally replace *in vivo* the *A. baumannii* 17978 BlsA protein and that the pathways leading to biofilm formation are inversely regulated at 24°C between these two microorganisms. Finally, we found the presence of predicted genes coding BLUF-containing proteins in all *Acinetobacter* sequenced genomes, even though the copy number is variable among them. Phylogenetic analysis suggests a common origin for all BLUF domains present in members of this genus, and could distinguish well-differentiated clusters that group together BLUF homologs from different species, a situation particularly clear for members of the ACB complex. Despite a role played by these BLUF domain-containing proteins in the photoregulation observed in the members of the genus *Acinetobacter* is a likely scenario given our findings in *A. baumannii* and *A. baylyi*, further research will contribute to confirm this possibility.

## Introduction

The members of the genus *Acinetobacter* are strictly aerobic, oxidase negative, ubiquitous Gram-negative cocobacilli that are frequently found in the environment but also in the hospital setting, where some particular groups of the genus have been associated with outbreaks of nosocomial infections [1]. Currently, the genus comprises 27 species with valid names (www.bacterio.cict.fr/a/acinetobacter.html) and several putative species with provisional designations including nine genomic species delineated by DNA-DNA hybridization [1], [2]. The *Acinetobacter calcoaceticus*-*Acinetobacter baumannii* (ACB) complex is a subgroup within the genus comprised by closely related species including *A. baumannii*, *A. calcoaceticus, Acinetobacter nosocomialis* and *Acinetobacter pittii* [1], [2]. *A. calcoaceticus* is mainly an environmental microorganism rarely involved in human infections, while *A. nosocomialis* and *A. pittii* are predominantly isolated from hospitalized patients [1]–[3]. *A. baumannii* is the clinically most important species of the genus. During the last decades, it has emerged as one of the most common opportunistic pathogens involved in hospital-acquired infections, generally affecting immunocompromised and severely injured patients [4]. Outbreak strains of *A. baumannii* are generally multidrug-resistant and can readily colonize nosocomial environments and withstand unfavorable conditions such as desiccation, nutrient starvation, and antimicrobial treatment. A key determinant for the success of this pathogen in clinical settings has been proposed to be its ability to attach to and form biofilms on abiotic and biotic surfaces [5]–[8]. We have recently reported that this nosocomial pathogen perceives light as a stimulus governing many processes of its life cycle at low environmental temperatures [5]. In fact, we found that blue light inhibited the formation of biofilms and pellicles in *A. baumannii* ATCC 17978 cells cultured at 24°C in liquid broth, and enhanced the ability of the bacteria to kill the filamentous form of the eukaryotic fungus *Candida albicans* [5]. We also showed that *A. baumannii* ATCC 17978 cells ceased moving on semisolid media plates in the presence of blue light at 24°C, while they spread all over the surface of the plates in the dark. Genome sequence analysis suggested a likely candidate for the photoreceptor implicated in this process: a BLUF-domain containing protein encoded by the A1S_2225 open reading frame. Its involvement in *A. baumannii* photoregulation was latter confirmed by both biophysical and genetic approaches, and therefore it was designated BlsA for *blue light sensing A* [5]. Interestingly, light regulation was lost at 37°C, a temperature compatible with warm-blooded hosts [5]. This temperature-dependency prompted speculations on the role of light sensing in the lifestyle of *A. baumannii*. Our current hypothesis postulates that light sensing may play a role during the pathogeńs environmental life, modulating its behavior outside the human body.

Many questions arise from our previous work, such as whether blue light regulation is widely distributed within the genus *Acinetobacter*. In such a case, relevant questions would be whether environmental species show light-mediated responses and temperature dependence similar to that described above in *A. baumannii*. Alternatively, blue light regulation could be restricted only to a subgroup in which *A. baumannii* is contained, suggesting that acquisition of light sensing genes occurred by recent horizontal gene transfer events.

Therefore, in this work we evaluated whether other species within the genus *Acinetobacter* also share the light-dependent responses described in *A. baumannii*, in particular, by studying photoregulation of motility and biofilm formation. We also provide insights into the evolution of BLUF-domains encoded within *Acinetobacter* genomes, shown to mediate light regulation in *A. baumannii*, by inferring and analyzing their phylogenetic relationships. This knowledge may contribute to our current understanding of the role, importance and evolution of light sensing and regulation in these bacterial species.

## Materials and Methods

### Bacterial Strains

The *A. baumannii* and *E. coli* strains, as well as the plasmids constructed and/or used in this work are listed in Table 1. The *Acinetobacter* strains used in this study, which include representatives of 25 validly named species, and their origin (if known) are listed in Table 2. ‘*A. indicus*’ and ‘*A. oleivorans*’ are effectively but not validly published names for single strains (CCM 7832 and DR1, respectively), and therefore are mentioned between apostrophes. Many of these strains have been reported in literature before while others, selected from our collection, have been identified at the species level by amplifed ribosomal DNA restriction analysis (ARDRA) [9]. All this information is also indicated in Table 2.

**Table 1:** Plasmids and strains (*A. baumannii* and *E. coli*) used in this study.

Strain/plasmid	Relevant characteristics^a^	Source/reference
Strains		
*A. baumannii*		
17978	Clinical isolate	ATCC
17978.OR	*blsA*::*aph* derivative of 17978; Km^R^	[5]
17978.ORp	17978.OR harboring pWH1266; Km^R^, Amp^R^	[5]
17978.ORc_BlsA_	17978.OR harboring pWHBLSA; Km^R^, Amp^R^	[5]
17978.ORc_1499_	17978.OR harboring pWH1499; Km^R^, Amp^R^	This work
17978.ORc_2110_	17978.OR harboring pWH2110; Km^R^, Amp^R^	This work
17978.ORc_2125_	17978.OR harboring pWH2125; Km^R^, Amp^R^	This work
17978.ORc_2129_	17978.OR harboring pWH2129; Km^R^, Amp^R^	This work
***E. coli***		
**DH5α**	**Used for DNA recombinant methods**	**Gibco-BRL**
**Plasmids**		
pGem-T	PCR cloning vector; Amp^R^	
pWH1266	*E. coli*-*A. baumannii* shuttle vector; Ap^R^, Tc^R^	[13]
pWHBLSA	pWH1266 harboring a wild type copy of *blsA* expressed under its own promoter; Amp^R^	[5]
pWH1499	pWH1266 harboring a wild type copy of ACI1499expressed under its own promoter; AmpR	This work
pWH2110	pWH1266 harboring a wild type copy of ACI2110expressed under its own promoter; Amp^R^	This work
pWH2125	pWH1266 harboring a wild type copy of ACI2125 expressed under its own promoter; Amp^R^	This work
pWH2129	pWH1266 harboring a wild type copy of ACI2129expressed under its own promoter; Amp^R^	This work

a Amp^R^, ampicillin resistance; Km^R^, kanamycin resistance; Tc^R^, tetracycline resistance.

**Table 2:** Blue light and temperature (24°C vs. 37°C) regulation of motility and biofilm formation by *Acinetobacter* strains studied in this work.

Species/Strain	Origin^a^	Reference	BLUF-containinggenes^b^	Motility				Biofilm formation^c^	
				24°C		37°C		24°C	
				L	D	L	D	L	D
***A. baumannii***									
ATCC 17978^T^ (**)	cerebrospinal fluid	[27]	1	–	+	+	+	–	+
17978.ORp				+	+	ND	ND	+(#)	+(#)
17978.ORc_1499_				–	+	ND	ND	–	+(#)
17978.ORc_2110_				±	+	ND	ND	–	+(#)
17978.ORc_2125_				–	+	ND	ND	–	+(#)
17978.ORc_2129_				–	+	ND	ND	–	+(#)
17978.ORc_BlsA_				–	+	ND	ND	–	+(#)
ATCC 19606^T^			1	–	–	–	–	+	+
Ab244		[4]		–	–	ND	ND	–	±
***A. baylyi***									
ADP1	soil	[28]	4	±	+	±	+	+	–
***A. beijerinckii***									
CCUG 56139	air sacculitis (horse)	[21]		–	–	ND	ND	±	–
NIPH 838^T^	wound	[21]		–	–	ND	ND	–	–
NIPH1065	toe-web	[21]		–	–	ND	ND	±	–
***A. bereziniae***									
ACI 449	soil	((^∧^)		–	–	–	–	+	±
ACI 552	unknown	((^∧^)		–	–	ND	ND	±(*)	±(*)
LMG988^T^	wound	[29]		–	–	ND	ND	+(*)	+(*)
***A. bouvettii***									
DSM 14964^T^	activated sludge plants	[30]		–	–	–	–	–	–
***A. brisouii***									
CCUG 61636^T^	peat	[31]		–	–	ND	ND	±	–
***A. calcoaceticus***									
ACI 412	soil	(^∧^)	2 (^∧∧^)	–	+	–	+	+	±
ACI 27	soil	(^∧^)		–	–	–	–	–	–
PHEA-2 (**)	waste water	[32]	2						
ACI 23	sputum	(^∧^)		–	–	–	–	+	+
LMG 1046^T^	soil	[27]		–	–	ND	ND		
***A. gerneri***									
DSM 14967^T^	activated sludge plants	[30]		–	–	–	–	+	±
***A. guillouiae***									
ACI 46	urine	(^∧^)		–	–	ND	ND	±	–
ACI 47	wound	(^∧^)		–	–	ND	ND	+(*)	+(*)
LMG1003^T^	wound	[29]		–	–	ND	ND	+(*)	+(*)
CUGG 50621	unknown			–	–	ND	ND	–	–
***A. gyllenbergii***									
NIPH 975	tracheal exudate	[21]		–	–	ND	ND	+	+
NIPH 822	wound	[21]		–	–	ND	ND	+	+
NIPH 230/CCUG56138	vagina	[21]		–	–	–	–	+	+
NIPH 2150^T^	urine	[21]		–	–	ND	ND	+	+
***A. haemolyticus***									
ACI 25	air	(^∧^)		–	–	ND	ND	±	±
ACI 31	pus	(^∧^)		–	–	ND	ND	±	±
ACI 927	unknown	(^∧^)		–	–	ND	ND	–	–
ACI 928	unknown	(^∧^)		–	–	ND	ND	–	–
CCM 2358^T^	sputum	[27]		–	–	ND	ND	–	–
***’A. indicus’***									
CCM 7832	soil	[33]		–	–	ND	ND	–	–
***A. johnsonii***									
LMG 999^T^	duodenum	[27]		–	–	ND	ND	±	–
ACI 166	unknown	(^∧^)		–	–	ND	ND	–	–
ACI 197	unknown	(^∧^)		–	–	ND	ND	–	–
SH046/CCUG 57820(**)	perineum	[34]	1	–	–	ND	ND	–	–
***A. junii***									
LMG 998^T^	urine	[27]		–	–	ND	ND	±	±
DSM 14968^T^(***)	activated sludge plants	[30]		–	–	ND	ND	–	–
ACI 191	unknown	(^∧^)		–	–	ND	ND	–	–
ACI 282	unknown	(^∧^)		–	–	ND	ND	–	–
***A. lwoffii***									
ACI 26	blood	(^∧^)		–	–	ND	ND	–	–
LMG 985	gangrenous lesion	[27]		–	–	ND	ND	±	–
LMG 1029^T^	unknown	[27]		–	–	ND	ND	±	–
ACI 172	unknown	(^∧^)		–	–	ND	ND	±	–
ACI 174	unknown	(^∧^)		–	–	ND	ND	–	–
SH145/CCUG 57819)(**)	hand	[34]	2	–	–	ND	ND	±	–
***A. nosocomialis***									
ACI 32	urine	(^∧^)	2(^∧∧^)	–	+	–	+	±	–
ACI 57	skin front	(^∧^)		–	–	ND	ND	±(*)	±(*)
ACI 911	unknown	(^∧^)		–	–	ND	ND	+	+
LMG 10619^T^	sputum	[2]		–	+	–	+	+	±
RUH 2624 ( = CCUG 57817)(**)	Forehead skin	[2]	3	–	–	ND	ND	+	±
***’A. oleivorans’***									
**DR1** (**)	rice paddy	[35]	2	–	+	–	+	+	+
***A. parvus***									
NIPH 384^T^	ear	[36]		–	–	ND	ND	–	–
NIPH 399	eye	[36]		–	–	ND	ND	–	–
***A. pittii***									
ACI 37	wound	(^∧^)		–	–	ND	ND	+	+
ACI 38	cerebrospinal fluid	(^∧^)		–	–	ND	ND	±(*)	±(*)
LMG1035^T^	cerebrospinal fluid	[2]		–	–	ND	ND	+(*)	+(*)
ACI 988	unknown	(^∧^)	2(^∧∧^)	–	+	±	+	±	–
SH024/CCUG 57818 (**)	axilla	[2]	2	–	–	ND	ND	+	±
***A. radioresistens***									
ACI 49	urine	(^∧^)		–	–	ND	ND	–	–
ATCC 43998^T^	cotton tampon	[37]		–	–	–	–	–	–
ACI 62	hospital pillow	(^∧^)		–	–	ND	ND	±(*)	±(*)
ACI 183	unknown	(^∧^)		–	–	ND	ND	±(*)	±(*)
SH164/CCUG 57822(**)	forehead	[34]	4	–	–	ND	ND	–	–
SK8 (**)	unknown		6						
***A. rudis***									
CCUG 57889^T^	raw milk	[38]		–	–	ND	ND	+	–
***A. schindleri***									
NIPH 883	urine	[39]		–	–	ND	ND	–	–
NIPH 1034^T^	urine	[39]		–	–	ND	ND		
ACI 940	unknown	(^∧^)		–	–	ND	ND	–	–
***A. tandoii***									
DSM 14670^T^	activated sludge plants	[28]		–	–	ND	ND	–	–
NIPH 2309	non-medical environment	(!)		–	–	ND	ND	±	±
***A. tjernbergiae***									
DSM 14971^T^	activated sludge plants	[30]		–	–	ND	ND	+	+
7B02	activated sludge plants	[30]		±	+	±	+	–	–
***A. towneri***									
DSM 14962^T^	activated sludge plants	[30]		–	–	ND	ND	–	–
***A. ursingii***									
NIPH 137^T^	blood	[39]		–	–	ND	ND	±	–
NIPH 840	urine	[39]		–	–	ND	ND	±	–
NIPH 841	blood	[39]		–	–	ND	ND	+	+
NIPH 842	urine	[39]		–	–	ND	ND	+	+
ACI 941	unknown	(^∧^)		–	–	ND	ND	–	–
***A. venetianus***									
LMG 19082^T^	taron beach	[40]		–	–	ND	ND	±	–
CCUG 60049	blood of *Dermochelyscoriacea* (turtle)	[41]		–	–	ND	ND	–	–
T4	Sea water	[40]		–	–	ND	ND	–	–

a If not indicated otherwise, strains are of human origin.

b Number of BLUF-containing genes deduced from the available sequenced genomes.

c All biofilms correspond to wall biofilms unless stated.

T Type strains.

(^∧^) These strains have been identified by ARDRA.

(!) These strains have been unambiguously identified by *rpoB* sequencing.

(*) Only pellicle biofilm formation.

(**) Strains whose genomes have been sequenced.

(***) The type strain of *A. grimontii*, a junior synonym of *A. junii*.

(#) Wall and pellicle biofilm formation simultaneously.

(^∧∧^) The presence of BLUF-coding genes was determined by amplification using specific primers and posterior sequencing. See Materials and Methods for details.

ND, not determined.

### Cell Motility Experiments and Biofilm Assays

Cell motility was tested on “swimming agarose” (Tryptone 1%, NaCl 0.5%, agarose 0.3%; 5) or LB agarose (Peptone 1%, NaCl 1%, yeast extract 0.5%, agarose 0.3%) plates incubated in the presence or absence of light. “Swimming agarose” is an inherited name for plates of this composition, but it should be noted that members of the genus *Acinetobacter* do not perform flagella-mediated swimming as they do not produce this type of cell appendage [10]. Biofilm assays were carried out in glass tubes as described before [5]. All assays were performed at least in triplicate for both light and dark conditions.

Plates or biofilm tubes were incubated at 24°C or 37°C in the dark or under light emitted by LED (light-emitting diode) arrays with an intensity of 6 to 10 µmol photons/m2/s, determined using a Li-250A Light Meter (LI-COR). Each array was built using three-LED module strips (containing three LEDs each) emitting blue, green, or red light with emission peaks centered at 462 nm, 514 nm, and 636 nm, respectively, as determined using a LI-COR LI-1800 spectroradiometer [5]. It should be noted that temperature of both the liquid or agar medium under the illumination conditions used in these experiments did not vary significantly from that measured under dark conditions or in the incubation chamber.

For quantification of biofilms assays, duplicate cultures for each sample at each condition were used. One tube was sonicated immediately for 5 s with a thin probe and the OD_600_ of the culture was determined to estimate total cell biomass. The supernatant of the other tube was aspirated and rinsed thoroughly with distilled water. The cells attached to the tube walls were visualized and quantified by staining with crystal violet and solubilization with ethanol–acetone as described in ref. [11]. The OD_580_/OD_600_ ratio was used to normalize the amount of biofilm formed to the total cell content of each sample tested, to avoid variations due to differences in bacterial growth under different experimental conditions. Error bars show standard error of the mean for 3 different biological replicates (n = 3).

### General DNA Procedures

Genomic and plasmid DNA were isolated as described before [4], and DNA restriction and cloning experiments were carried out using standard protocols [12]. DNA sequences were determined at the DNA Sequencing Facility of the University of Maine, Orono, ME, USA; or at Macrogen (Korea).

### Construction of Complementation Plasmids

DNA fragments of 886, 890, 942 and 772 bp, corresponding to *A. baylyi* ACIAD1499, ACIAD2110, ACIAD2129, and ACIAD2125 predicted BLUF domain-containing genes and cognate promoters were amplified by PCR using *A. baylyi* strain ADP1 total DNA and primers 1499F (5′-*ggatcc*ctcatcaactataagta-3′) and 1499R (5′-*ggatcc*aagtggctgatattaat-3′), 2110F (5′-*ggatcc*acctcataacagtgtat-3′) and 2110R (5′-*ggatcc*tatttatgatccatcta-3′), 2125F (5′-*ggatcc*taacgacaagctataat-3′) and 2125R (5′-*ggatcc*acatatgaaagatacat-3′), 2129F (5′-*ggatcc*agatatgtactcactca-3′) and 2129R (5′-*ggatcc*gattatgtactggtaga-3′), all of which were tailed with BamHI restriction sites (indicated in italics in the primer sequences). The corresponding amplicons were first cloned into pGEM-T Easy (Promega) and then subcloned as BamHI fragments into the cognate site of pWH1266 [13]. Proper constructions of the complementing plasmids were verified by automated DNA sequencing. Plasmid DNA was electroporated into *A*. *baumannii* ATCC 17978 *blsA* mutant (17978.OR) as described before [5].

### Amplification and Sequencing of BLUF-coding Genes

The presence of BLUF domain-coding genes in strains *A. calcoaceticus* ACI 412, *A. nosocomialis* ACI 32 and *A. pittii* ACI 988, was analyzed by colony PCR using sequence information derived from the genome-sequenced strains *A. calcoaceticus* PHEA-2, *A. nosocomialis* RUH 2624 and *A. pittii* SH 024 to design specific primers that amplify the cognate BLUF genes: *A. calcoaceticus* ADY82057, primers 82057F 5′-ggACATATgTATgCAAgTAAAACCA-3′ and 82057R 5′- ggATCCTTAACCTTgATATTgATCA-3′; *A. nosocomialis* EEW98085, primers 98085F 5′- gCTAgCATgAATgACTTTAgACTAC-3′ and 98085R 5′- ggATCCTTACTTTTTTAAAgCTTTACT-3′; *A. nosocomialis* EEX00046, primers 46F 5′- ggACATATgAgTTTAATAggCTTTATg-3′ and 46 R 5′- ggATCCTTAAACTTgATATTgATCCg-3′, and *A. pittii* EFF86081, primers 86081F 5′- ggACATATgAgTTTAATAggCTTTATg-3′ and 86081R 5′- ggATCCCTAACCTTgATATTgATCAA-3′). To amplify *A. baumannii* BlsA close homologs present in these strains (ADY82317, EEX01065 and EFF86339), we used primers EblsA.R/5 (5′-GGATCCCTAGAACGGGTTTAC-3′) and EblsA.F/6 (5′-CATATGAACGTTCGCCTGTGT-3′) previously described in ref. [5]. The amplified fragments were purified by gel extraction (GFX, Amersham) and sequenced by Macrogen (Korea).

### Disc Diffusion Antibiotic Susceptibility Test

Müeller Hinton (MH) agar plates were inoculated with a culture of each tested strain, which were previously adjusted to 0.5 McFarland standard turbidity using fresh culture medium or saline solution, according to the recommendations of the National Committee for Clinical Laboratory Standards [14]. Antimicrobial commercial discs (BBL, Cockeysville, MD, USA) containing 10 mg of ampicillin, 30 mg of amikacyn, 30 mg of cefepim, 30 mg of cefotaxime, 30 mg of cefoxitin, 30 mg of cephalotin, 30 mg of chloramphenicol, 5 mg of ciprofloxacin, 10 mg of imipenem, 10 mg of gentamycin, 10 mg of meropenem, 100 mg of piperacillin or 5 mg of rifampicin were placed on the surface of plates, which were latter incubated overnight at 24°C or 37°C under blue light or darkness. The assays were performed in triplicate.

### Sequence Analyses

Protein sequences were retrieved from databases at NCBI (http://www.ncbi.nlm.nih.gov) and Pfam [15], and were aligned using ClustalW version 1.7 [16]. To exclude the high sequence variability often found after BLUF domains which -in some cases- supposes diversity of the accompanying effector domains [17], only the region corresponding to the BLUF domain (comprising 93–96 amino acid residues, depending on the organism) was extracted from the complete sequences and used for the alignments. Gaps were removed from the alignments using BioEdit version 7.05.3 [18].

### Phylogenetic Inferences

Phylogenetic relationships were inferred from amino acid sequence alignments using the programs provided in the PHYLIP package, version 3.69 [19] (http://evolution.genetics.washington.edu/phylip.html). The maximum-likelihood method (PROTML) was used for the construction of the BLUF-domain phylogenetic tree. In all cases, confidence levels were calculated from 1,000 bootstrap resamplings (SEQBOOT) of alignments used for phylogenetic inferences by both neighbor-joining method using a Dayhoff PAM distance matrix (PROTDIST) and the parsimony (PROTPARS) method, also included in the PHYLIP software package [19].

## Results

### Motility is also Regulated by Blue Light in non-*A. baumannii* Members of the Genus *Acinetobacter*

**Figure 1:**
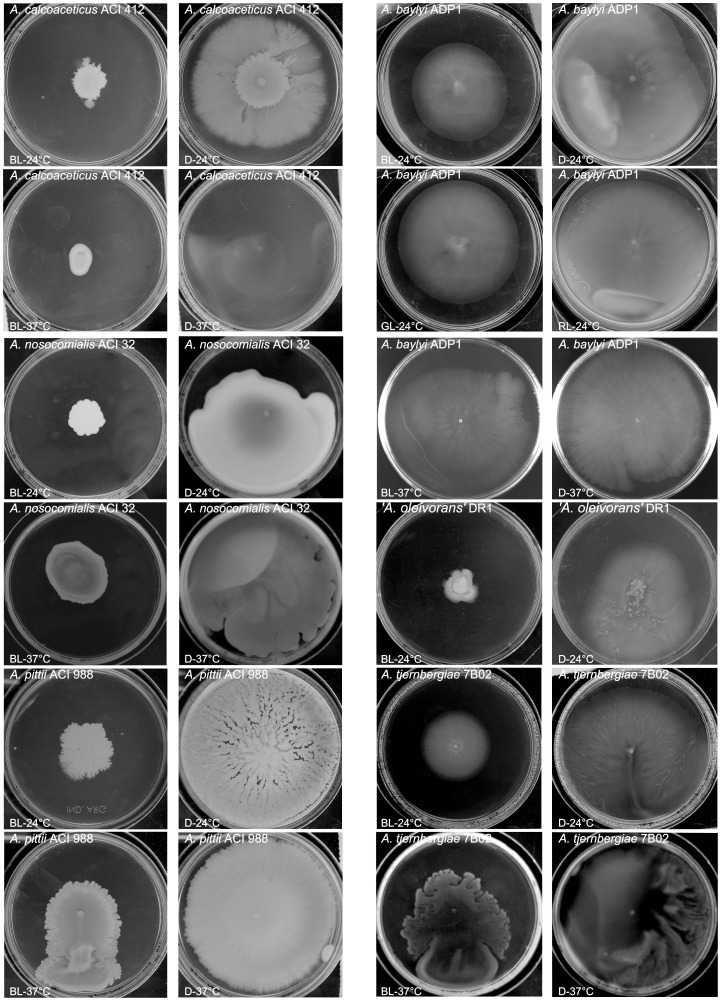
Effect of light and temperature on bacterial motility. Cells of different species within the genus *Acinetobacter* were inoculated on the surface of swimming plates. Plates were inspected and photographed after incubated overnight in darkness (D) or in the presence of blue light (BL), green light (GL) or red light (RL) at 24°C or 37°C. Only some strains displaying photoregulated motility are shown.

Given that light regulates motility in *A. baumannii* [5], we analyzed whether other species of this genus are also able to move and respond to light at 24°C. We found that *A. baylyi, A. calcoaceticus, A. nosocomialis, ‘A. oleivorans’, A. pittii* and *A. tjernbergiae* showed light-dependent regulation of motility, at least in one from three to five strains assayed (see Figure 1 and Table 2). Just as described for *A. baumannii* [5], motility in these bacteria was inhibited under blue light, while in the dark they spread all over the surface of the plate (Figure 1). In the case of *A. baylyi* and *A. pittii*, blue light inhibition was not absolute and bacteria were still able to move, even though to a much lesser extent than in the dark (Figure 1). It is interesting to note that the appearance of *A. pittii* is different from that observed for other species such as *A. baumannii* or *A. calcoaceticus. A. pittii* did not move homogeneously from the inoculation point, but rather formed independent striations irradiating from the center. We did not observe motility in any of the *A. beijerinckii, A. bereziniae, A. bouvettii, A. brissouii, A. gerneri, A. guillouiae*, *A. gyllenbergii, A. haemolyticus, ‘A. indicus’, A. johnsonii*, *A. junii*, *A. lwoffii*, *A. parvus*, *A. radioresistens*, *A. rudis*, *A. schindleri, A. tandoii, A. towneri, A. ursingii*, and *A. venetianus* strains analyzed, even though as much as five different strains were tested for several species (Table 2). In our previous study we showed that light regulation of motility in *A. baumannii* occurred at 24°C but not at temperatures associated with warm-blooded hosts such as 37°C [5]. We thus evaluated next whether any of the strains that showed photoregulation at 24°C also exhibited light regulation at 37°C and found that, in contrast to *A. baumannii,* all of them did respond to light at a higher incubation temperature (Figure 1 and Table 2). It should be noted that similar results were obtained when all strains were tested using LB agarose 0.3% instead of “swimming agarose” under similar experimental conditions.

### Biofilm Formation is also Regulated by Blue Light in non-*A. baumannii* Members of the Genus *Acinetobacter*

**Figure 2:**
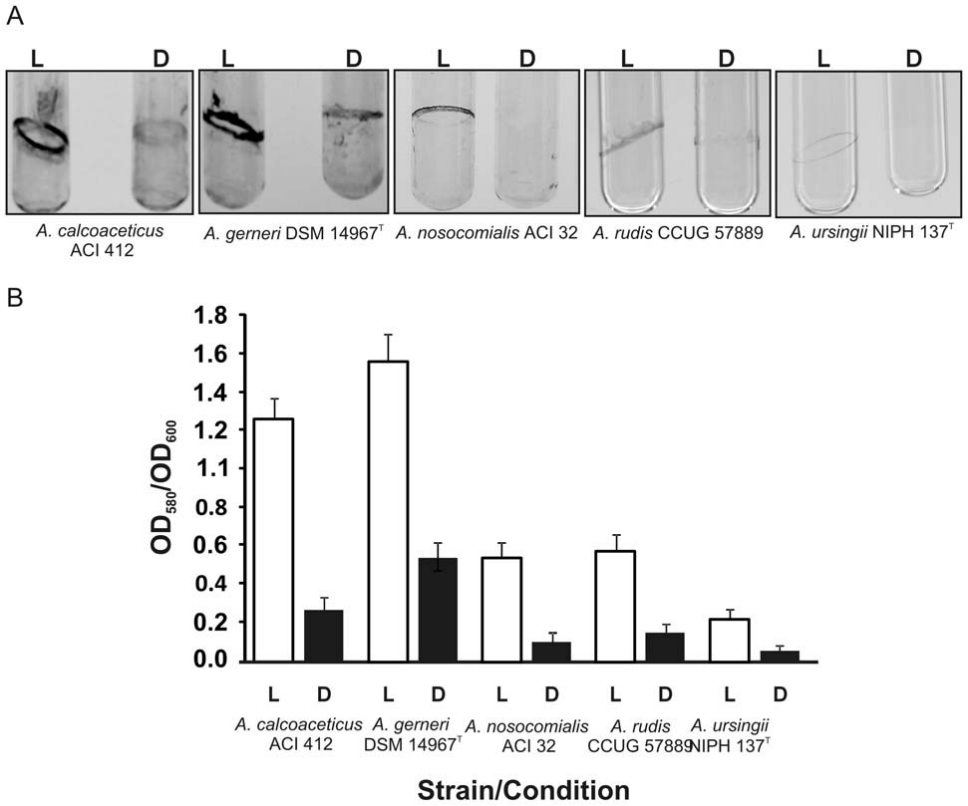
Effects of light on biofilm formation. A. The biofilms formed by cells of the different species within the genus *Acinetobacter* on glass tubes in the presence of blue light (L) or in darkness (D) were recorded after static incubation for 96 h at 24°C by direct visual inspection and staining with crystal violet. Only representative examples are shown. B. Quantification of the biofilms of cognate samples shown in A. Error bars show standard error of the mean for 3 different biological replicates (n = 3). OD_580_/_600_, optical density at 580 or 600 nm, respectively. L: light; D: Dark.

We further evaluated whether biofilm formation was photoregulated in different species within the genus by studying their ability to form biofilms on glass both under blue light and in the dark at 24°C. We found that at least one strain of *A. baylyi, A. bereziniae, A. calcoaceticus, A. gerneri* and *A. rudis* formed large amounts of biofilm on tubes incubated stagnantly under blue light for four days, while the levels of biofilms formed in the dark were significantly lower or negligible (Figure 2, Figure S1 and Table 2). Some strains of *A. beijerinckii, A. brissouii, A. guillouiae, A. johnsonii, A. lwoffii, A. nosocomialis, A. pittii, A. ursingii*, and *A. venetianus* also showed photoregulation of biofilm formation (Figure 2, Figure S1 and Table 2), but the levels of wall biofilms were lower than in the aforementioned strains (Figure 2A). It is interesting to note that even though there is photoregulation of biofilm formation in these species (similarly to *A. baumannii*), the amount of wall biofilms formed by non-*A. baumannii* species was much greater under blue light than in darkness, in contrast to *A. baumannii* in which larger levels are observed in the dark [5]. In non-*A. baumannii* species, we observed mainly the presence of wall biofilms only. The presence of pellicles (with no wall biofilm occurring at the same time) was evident only in a few strains of *A. bereziniae*, *A. guillouiae*, *A. nosocomialis*, *A. pittii* and *A. radioresistens,* and no light regulation was detected on them. The effect of light on biofilm formation at 37°C by the *Acinetobacter* strains used in this study could not be evaluated because of wide variations observed in different assays. Such a response is not surprising, as similar situations have been described before for clinical isolates of *A. baumannii* [5]. A summary of the biofilm properties of all the strains studied is presented in Table 2, and quantification of biofilm formation for strains showing photoregulation is shown in Figure 2 for representative cases or Figure S1.

### *A. baylyi*: a Case Study

The fact that *A. baylyi* harbors four paralogous genes encoding BLUF-containing putative photoreceptors, designated ACIAD1499, ACIAD2110, ACIAD2125 and ACIAD2129 (Table 2), suggests that light might play a key role in the lifestyle of the bacterium, which could justify the abundance of these genes and their possible functional redundancy. To better characterize the response of this bacterium to light, we compared its ability to move under red or green light with its response under blue light or darkness at 24°C. We observed that green light inhibited motility, even though to a lesser extent than blue light (Figure 2). In contrast, under red light the bacteria behaved as in darkness. Thus, *A. baylyi* is able to sense and respond to green light in a similar way as *A. baumannii* [5], indicating that at least one of the four putative photoreceptors is capable to respond to green light.

We assayed next whether any of the different photoreceptors present in *A. baylyi* were able to rescue the lost photoregulation of motility and biofilm formation at 24°C of the *A. baumannii* ATCC 17978 *blsA* mutant (17978.OR) [5]. For this purpose, we cloned each of the *A. baylyi* predicted photoreceptor genes under their own promoter control in the shuttle plasmid pWH1266 to generate pWH1499, pWH2110, pWH2125 and pWH2129 (Table 1). Then, we transformed the 17978.OR mutant strain with these constructions, as well as with the empty vector, to generate 17978.ORc_1499_, 17978.ORc_2110_, 17978.ORc_2125_, 17978.ORc_2129_ and 17978.OR_p_, respectively. Finally, we analyzed whether these strains showed photoregulation of motility or biofilm formation at 24°C.

**Figure 3:**
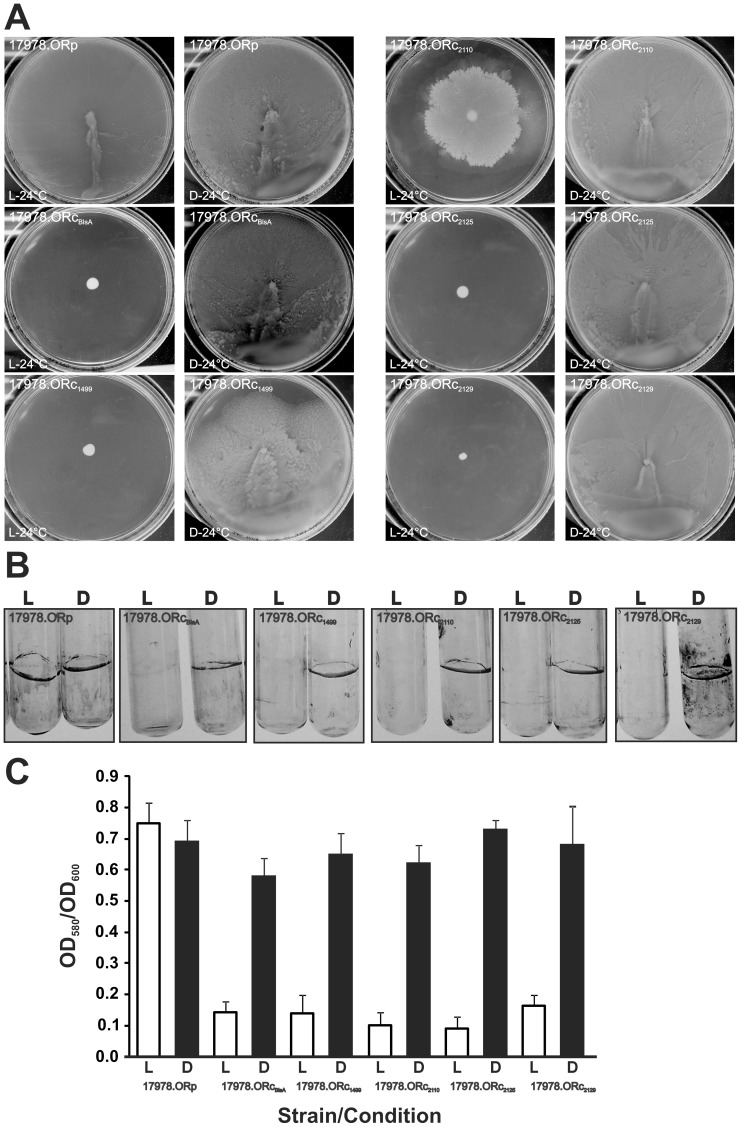
Effect of blue light on biofilm and motility mediated by *A. baylyi* ´s photoreceptors in an *A. baumannii* genetic background. A. Cells of the ATCC 17978.OR *blsA* mutant, transformed with plasmids pWH1499, pWH2110, pWH2125 and pWH2129 or the empty pWH1266 vector, were inoculated on the surface of swimming plates. Plates were inspected and photographed after overnight incubation in darkness (D) or the presence of blue light (L) at 24°C. B. The biofilms formed by ATCC 17978.OR *blsA* mutant, transformed with plasmids pWH1499, pWH2110, pWH2125 and pWH2129 or the empty pWH1266 vector on glass tubes were recorded after static incubation for 96 h at 24°Cby direct visual inspection and staining with crystal violet. C. Quantification of the biofilms of cognate samples shown in B. Error bars show standard error of the mean for 3 different biological replicates (n = 3). OD_580_/_600_, optical density at 580 or 600 nm, respectively. L: light; D: Dark.

Figure 3A shows that 17978.OR harboring the empty pWH1266 plasmid (17978.ORp) does not exhibit photoregulation of motility, spreading throughout the plate either under blue light or in the dark at 24°C. In contrast, 17978.ORc_1499_, 17978.ORc_2125_ and 17978.ORc_2129_ showed a tight inhibition of motility under blue light while spreading throughout the plate in the dark. Such response resembles that of the complemented 17978.ORc_BlsA_strain harboring the native *A. baumannii blsA* wild-type allele (see Figure 3A) [5]. Therefore, the corresponding *A. baylyi* putative photoreceptors are able to fully complement the *blsA* gene. Conversely, the *A. baylyi* photoreceptor encoded by ACIAD2110 only partially complemented the *blsA* mutation in ATCC 17978 showing that it is not able to fully restore BlsA functioning in *A. baumannii*, at least regarding motility (Figure 3A).

We also analyzed the effects of the expression of the different *A. baylyi* photoreceptors on the ability of the *A. baumannii* ATCC 17978.OR cells to form biofilms at 24°C (Figure 3B and C). In this case, all four *A. baylyi* BLUF-domain containing photoreceptors restored the original phenotype, behaving as the 17978.ORc_BlsA_ strain when tested as described before [5].

### Blue Light and Resistance to Antibiotics

As many clinically-relevant species of *Acinetobacter* show an outstanding ability to rapidly evolve resistance to antibiotics, reducing therefore the available therapeutic options [4], we speculated whether blue light also modulates resistance to antibiotics. For this purpose, we conducted disc-diffusion antibiotic susceptibility assays both at 24°C as well as 37°C under blue light or in the darkness, using strains *A. nosocomialis* ACI 32, *A. pitii* ACI 988, both of which show photoregulation of motility and biofilm formation, and *A. haemolyticus* ACI 25. Despite various antibiotics belonging to different groups such as ampicillin (β-lactam), ceftazidime (β-lactam), cephalotin (β-lactam), chloramphenicol (chloramphenicol), ciprofloxacin (fluoroquinolone), gentamycin (aminoglycoside), imipenem (carbapenem β-lactam), meropenem (carbapenem β-lactam), piperacillin (β-lactam) and rifampicyn (rifamycin) were tested, no significant differences were detected between light and dark conditions at either temperature for these strains (Table S1).

We also evaluated the effect of light on antibiotic resistance in *A. baumannii* strains, as this species is the most frequently recovered in clinical settings and the one that has been extensively reported to exhibit resistance to multiple antibiotics [4]. We used here strain ATCC 17978 (as it has been reported to respond to light), which is sensitive to most antibiotics; as well as strain Ab244 [4], which shows resistance to multiple antibiotics and a slight photoregulation of biofilm formation (Figure S1). Here again, although many antibiotics belonging to different groups were tested, e.g., imipenem, meropenem, cloramphenicol, rifampicin, ampicillin, amikacyn (aminoglycoside), piperacillin, cefoxitin (β-lactam), cephalotin (β-lactam), cefotaxime (β-lactam), cefepim (β-lactam) and ceftazidime (β-lactam), no significant differences were observed that could result from differential resistance mediated by light (Table S1).

### BLUF-domain Containing Proteins in non-*A. baumannii* Members of the Genus *Acinetobacter*

We showed previously that the BLUF domain-containing protein encoded in the *A. baumannii* ATCC 17978 genome is an active photoreceptor that modulates different traits such as motility and biofilm formation [5], and also that the four BLUF domain-containing proteins encoded in *A. baylyi* strain ADP1 are active photoreceptors able to sense light and transduce the signal in the *A. baumannii* genetic background modulating motility and biofilm formation in this organism. To further extend our knowledge of BLUF domain-containing proteins, we analyzed the presence and phylogenetic relationships of these domains in other members of the genus *Acinetobacter*, as they could likely play similar roles in other species. For this purpose, we screened the complete (or almost complete) sequenced genomes available in databases of members of the genus *Acinetobacter* for the presence of genes coding for BLUF-domain containing proteins, to determine if their presence is distributed in the genus and evaluate their phylogenetic relatedness. Genes coding for BLUF-containing proteins were present in all of the screened genomes, i.e. those of *A. baumannii*, *A. baylyi*, *A. calcoaceticus*, *A. johnsonii*, *A. lwoffii*, *A. nosocomialis*, ‘*A. oleivorans*’, *A. pittii*, *A. radioresistens*, and *Acinetobacter* sp. ATCC 27244 (Table 2). It is worth mentioning that based on the Pasteur MLST scheme [20], as well as *rpoB* sequence comparisons and phenotypic analyses [21], ‘*A. oleivorans*’ DR1 is highly related to one of the two strains designated ‘Between 1 and 3′, being therefore also a member of the ACB complex [A. Nemec, unpublished data].

As shown in Table 2, the number of predicted BLUF-containing proteins encoded per genome in the above species fluctuates from one to six. Indeed, it is noteworthy that close species such as those comprised within the ACB complex show variability in the number of genes coding for BLUF-domain containing proteins. As seen in Table 2, *A. calcoaceticus* PHEA-2, *A. pittii* SH024 and ‘*A. oleivorans*’ DR1 encode two, while *A. baumannii* and *A. nosocomialis* RUH2624 encode one and three BLUF-domain containing proteins, respectively. Besides, *A. baylyi* ADP1 encodes four putative BLUF-photoreceptors while *A. radioresistens* SK8 and SH164 encode six and four, respectively. All of the predicted BLUF-proteins found in members of the genus *Acinetobacter* correspond to the most common bacterial BLUF photoreceptors, small proteins containing a flavin-binding photosensing core lacking a recognizable effector or output domain(s), such as BlsA from *A. baumannii* [5].

**Figure 4:**
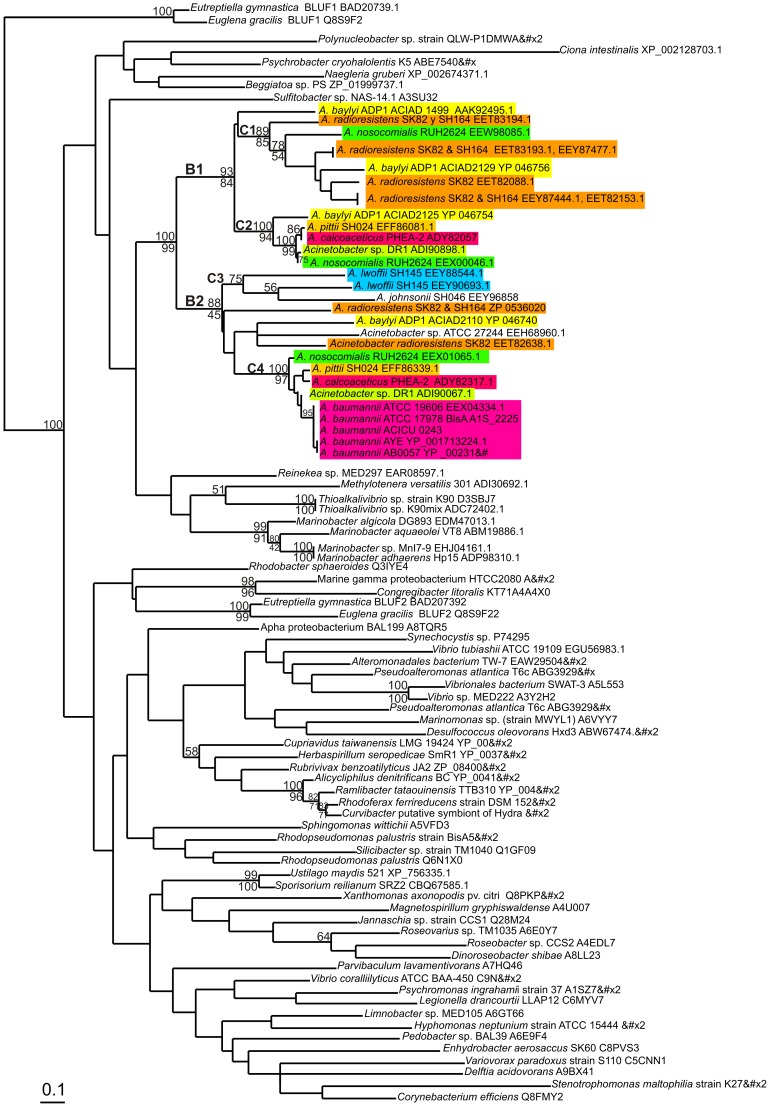
Phylogenetic relationships of BLUF domains found in different taxa. Maximun likelihood phylogenetic tree of BLUF domain constructed using sequences from different branches of Bacteria, Euglenozoa and Fungi. The accession numbers of the different sequences are indicated after the corresponding species names. The different clusters supported by bootstrap values (C1 to C4) within *Acinetobacter* are indicated. Paralogs from the same *Acinetobacter* species are indicated with the same color. The percentages of 1,000 bootstrap resamplings supporting the different clusters, as obtained by Neighbor Joining (above the lines) or parsimony (below the lines), are indicated atthe bifurcations (only bootstrap results of >50% are reported).

To determine the phylogenetic relationships between the BLUF domains present within the *Acinetobacter* genus, we retrieved 93 protein sequences corresponding to the BLUF domains of predicted and known blue-light photoreceptors of different members of this genus, and also of organisms belonging to different taxa such as α, β, γ and δ Proteobacteria; and from eukaryotes such as Euglenozoa and Fungi (Figure 4) [22]. In some cases such as those of *Euglena* and *Eutreptiella*, which contain two BLUF-domains in the same protein molecule, both sequences were included in the analyses. Figure 4 shows the maximum likelihood phylogenetic tree constructed from the alignments of the above sequences, whereby bootstrap values were calculated by the neighbor joining and parsimony methods. The tree clearly illustrates that all the BLUF domains present in members of the genus *Acinetobacter* are grouped together in a well-supported monophyletic cluster (bootstrap values of 100% and 99% by NJ and parsimony, respectively), suggesting that all of these putative photoreceptor domains share a common origin. The *Acinetobacter* cluster contains two major branches, B1 and B2 (Figure 4). Each branch contains at least one paralog gene from each species (similar colors in B1 and B2), with the exceptions of *A. lwoffii* (whose paralogs are restricted to B2, light blue sequences) and all *A. baumannii* strains, each showing one gene coding for highly similar BLUF-domain containing proteins (*blsA*), also concentrated in B2.

It is worth noting that, with the only exception of *Psychrobacter*, the other genera of the Moraxellaceae family, *i.e. Alkanindiges*, *Enhydrobacter*, *Moraxella*, *Oligella*, *Paraperlucidibaca* and *Perlucidibaca,* do not contain genes coding for BLUF-containing proteins. Despite the presence of BLUF domains in *Psychrobacter*, as exemplified by the homolog present in *P. cryohalolentis*, this sequence does not group together with those of *Acinetobacter* (Figure 4), indicating significant divergence from BLUF domains present in members of this genus. Overall, no horizontal gene transfer between members of the *Acinetobacter* genus and other non-*Acinetobacter* BLUF-containing organisms is evident from the tree. Most likely, all BLUF-domain containing genes found in currently existing species of the *Acinetobacter* genus evolved from a aboriginal BLUF-domain containing gene already present in the common ancestor of the genus. Further analysis allows the differentiation of four clusters supported by bootstrap values (C1, C2, C3 and C4, Figure 4) that group together BLUF domain homologs from different species (particularly clear for members of the ACB complex). For example, cluster C4 within B2 groups BLUF domain sequences of members of the ACB complex such as all *A. baumannii* BlsA proteins, with homologs present in *A. calcoaceticus*, DR1, *A. nosocomialis* and *A. pittii*, which in turn share a monophyletic cluster with *A. baylyi* (ACIAD2110), *A. radioresistens*, and *Acinetobacter* ATCC 27244. Cluster C2 within B1 groups together another set of BLUF domain homologs also present in members of the ACB complex including *A. calcoaceticus*, DR1, *A. nosocomialis* and *A. pittii*, all of them composing a monophyletic cluster with *A. baylyi* BLUF domain (ACIAD2125). Finally, in cluster C1 (B1) are grouped together a third BLUF domain paralog present in *A. nosocomialis* RUH2624 sharing cluster with homologs present in *A. baylyi* (ACIAD1499 and ACIAD2129) and *A. radioresistens*. Overall, multiple duplications and differential gene losses occurring during the evolution of the different *Acinetobacter* lineages could explain the presence of different number of paralogs of BLUF-domain containing genes in the diverse species composing the genus. Besides, the homologs from different species grouped altogether in different clusters (C1–C4) could correspond to orthologs; i.e. they were separated by a speciation event, but also could have resulted from horizontal gene transfer of BLUF-coding genes among *Acinetobacter* species.

Finally, no robust bootstrap values where obtained for branches grouping non-*Acinetobacter* sequences, probably due to structural more than sequence similarities between the different BLUF domains.

### Presence of BLUF-coding Genes in Strains Showing Photoregulation of Motility and Biofilm Formation

Finally, we analyzed whether strains that presented photoregulation of motility and biofilm formation such as *A. calcoaceticus* ACI 412, *A. nosocomialis* ACI 32 and *A. pittii* ACI 988 contained BLUF domain-containing genes. For this purpose, we used information derived from the genome-sequenced strains *A. calcoaceticus* PHEA-2, *A. nosocomialis* RUH 2624 and *A. pittii* SH 024 to design specific primers that amplify the cognate BLUF coding genes (acc. numbers ADY82057 and ADY82317 for *A. calcoaceticus*; EEW98085, EEX00046 and EEX01065 for *A. nosocomialis*; and EFF86081 and EFF86339 for *A. pittii*), and investigated their presence by PCR and nucleotide sequencing. We found the presence of homologs showing 100% identity to ADY82057 and ADY82317, in *A. calcoaceticus* ACI 412, EEX00046 and EEX01065 in *A. nosocomialis* ACI 32, and EFF86081 and EFF86339 in *A. pittii* ACI 988 (Table 2). We were not able to obtain an amplification product in the case of *A. nosocomialis* EEW98085, despite we assayed different amplification conditions.

## Discussion

In this work, we show that light regulation is not restricted to *A. baumannii* but is rather widespread within the genus *Acinetobacter*. In fact, we found that blue light effectively regulates motility and biofilm formation at 24°C in many *Acinetobacter* species, including members of the ACB complex, such as *A. calcoaceticus, A. nosocomialis* and *A. pittii* (see Figure 1 and Table 2). Yet, in contrast to *A. baumannii*, in which the formation of biofilms is inhibited under blue light while stimulated in the dark, the opposite was observed in all the other species where blue light regulation was detected: biofilm formation was inhibited in the dark while stimulated under blue light. In non-*A. baumannii* species where both biofilm formation and motility were regulated such as *A. baylyi*, *A. calcoaceticus*, *A. nosocomialis* and *A. pittii,* low biofilm correlated with high motility under dark conditions. This response makes sense since a large amount of data support the notion that motility and biofilm formation can be expected to be mutually exclusive and counter-regulated, i.e. being sticky seems counterproductive for moving around, whereas adhesion and settling down might require reduced activity of the motility machinery [23]–[25]. Therefore, in these bacteria blue light contributes to the decision between motility and sessility and also may facilitate acclimation to different environments. In our previous work, we showed that blue light regulation in *A. baumannii* occurred only at low temperature, suggesting that it is important during its life in the environment perhaps allowing bacteria to sense environmental locations outside the human host [5]. However, the results presented here show that many environmental species such as *A. baylyi*, *A. calcoaceticus* and *A. tjernbergiae,* as well as the clinically relevant species *A. nosocomialis* and *A. pittii* displayed blue light regulation of motility also at 37°C. This differential behavior at 37°C compared to *A. baumannii* may result from the extra content of BLUF-domain putative photoreceptors encoded in the genomes of these non-*A. baumannii* species (Figure 4 and Table 2). Indeed, only one photoreceptor is encoded in the *A. baumannii* genome. The protein present in strain ATCC 17978, BlsA, most probably functions only at 24°C: *blsA* mRNA levels at 37°C are significantly lower with respect to levels at 24°C [5], and the content of BlsA protein in the cells at 37°C is negligible or null [Mussi *et al.*, unpublished data]. Alternatively, the differential behavior at 37°C between *A. baumannii* and other *Acinetobacter* species could result from idiosyncratic differences in expression patterns of photoreceptors and/or pathways and partners modulating motility functions downstream the photosensing step in these organisms. In any case, *A. baumannii* might have become “blind” to light at 37°C because there is no positive selection to respond to this stimulus in the relative darkness of the warm-blooded host tissues.

The ability of *A. baylyi*’s BLUF-domain containing genes to restore photoregulation at 24°C in the ATCC 17978 *blsA* mutant not only confirms that they encode bona fide BLUF photoreceptors, but also that they can transduce the light signal into *A. baumannii* motility and biofilm regulatory cascades, probably by using BlsA partner/s. In this context, *A. baylyi* formed large amounts of biofilms under blue light and almost negligible amounts were produced under dark conditions. It is therefore noteworthy that the opposite situation is observed when *A. baylyi* ´s photoreceptors are expressed in the *A. baumannii blsA* mutant, restoring in all cases the wild type phenotype corresponding to this species. Therefore, both cascades in *A. baumannii* and *A. baylyi* seem to be inversely affected at 24°C, independently of the origin of the photoreceptor used.

Moreover, we found that BLUF-domain containing genes, shown to be active photoreceptors in *A. baumannii* and *A. baylyi*, are present in all completely sequenced genomes available for members of this genus, and also in strains showing photoregulation of motility and biofilm formation, such as *A. calcoaceticus* ACI 412, *A. nosocomialis* ACI 32 and *A. pittii* ACI 988. The variable number of genes coding for BLUF-domain containing proteins (from one to six) in the different species analyzed in this study suggests that sensing and responding to light might be of differential importance among them, probably reflecting their different lifestyles and the diversity in niches in which they thrive. Phylogenetic analysis suggests a common origin for all BLUF domains within *Acinetobacter* and could distinguish well-differentiated clusters that group together BLUF homologs from different species, a situation particularly clear for members of the ACB complex, which most likely correspond to groups of orthologs. The different clusters may reflect a closer phylogenetic relationship among the species with related orthologs, or the observed clustering could have resulted from horizontal gene transfer of BLUF-coding genes among *Acinetobacter* species.

Finally, we were not able to detect photoregulation of neither motility nor biofilm formation in many species or strains of the genus *Acinetobacter*, despite the presence of multiple BLUF-domain containing genes (such as the 4–6 putative BLUF-domain photoreceptors present in the *A. radioresistens* genomes, Table 2). This is not surprising since a similar situation has been previously reported by us in the case of *A. baumannii* strain ATCC 19606^T^ [5]. Many possibilities could lead to the above results: the machineries of motility or biofilm formation may be impaired in some of these strains, or their specific components may not be expressed under the conditions studied. Still, light could be regulating other cellular processes not tested in this work. Besides, other possible explanations suppose that photoreceptor genes in these strains might not be expressed under the assayed conditions, the corresponding proteins may not be active, or downstream partners of the signaling cascade or targets of the photoreceptors involved in these phenotypes might be missing. Leaving aside particular cases, which would not be unexpected since a high genetic heterogeneity has been described for strains of some members of *Acinetobacter* such as *A. baumannii* [4], [5], [26], the conservation of multiple BLUF-domain containing genes as well as the fact that they have not been subjected to genetic derive, suggests that they have been maintained to play important roles in the bacterial physiology. Most probably, these putative photoreceptors could serve functions modulating other cellular processes that remain unidentified in these species. Yet, functional characterization of BLUF domain-containing proteins encoded in species of the genus *Acinetobacter* other than those of *A. baumannii* or *A. baylyi* still needs to be conducted to ascertain their role as photoreceptors involved in light perception in these microorganisms. Regarding other cellular processes affected by light, it is worth mentioning that we could not detect light regulation of resistance to antibiotics in different clinically-relevant species of the genus *Acinetobacter*. Nevertheless, further research would contribute to draw a final conclusion in this sense.

Our understanding of the signal transduction mechanisms and regulatory cascades involved in *A. baumannii* BlsA and its homologs present in other species of the genus *Acinetobacter* is still scarce and currently under study in our laboratory. The final goal is to gain a full comprehension of light regulation in relation to host’s niches and lifestyles, which would perhaps need further understanding of *Acinetobacter* biology.

## Supporting Information

Figure S1 **Quantification of the biofilms produced by different strains showing photoregulation of biofilm formation within the genus** ***Acinetobacter*** **.** Error bars show standard error of the mean for 3 different biological replicates (n = 3). OD_580_/_600_, optical density at 580 or 600 nm, respectively.
(TIF)

Table S1 **Blue light and resistance to antibiotic.** Disc difussion antibiotic susceptibility assay under blue light or in the dark at 24 or 37°C, of some strains of *A. nosocomialis*, *A. pittii* and *A. baumannii* which showed photoregulation of motility and/or biofilm formation. The *A. haemolyticus* strains analyzed in this work did not show photoregulation neither of motility nor biofilm formation, but one strain was included in this study due to the importance of this species in the clinical settings. The diameter of inhibition from three independent experiments (mm +/− SEM of three biological replicates) is indicated. AM, ampicillin; AN, amikacyn; FEP, cefepim; CTX, cefotaxime; FOX, cefoxitin; CAZ, ceftazidime; CF, cephalotin; C, chloramphenicol; CIP, ciprofloxacin; IPM, imipenem; GM, gentamycin; MEM, meropenem; PIP, piperacilin; RA, rifampicin.
(XLSX)

## References

[1] Vaneechoutte M, Kaempfer P, Dijkshoorn L, Nemec A, Wauters G (2011) *Acinetobacter*, *Chryseobacterium*, *Moraxella*, and other nonfermentative Gram-negative rods. In: Versalovic J, Carroll KA, Funke G, Jorgensen JH, Landry ML, Warnock DW, editors. Manual Clinical Microbiology 10^th^ Ed. Washington DC: ASM Press. 714–738.

[2] Nemec A, Krizova L, Maixnerova M, van der Reijden TJ, Deschaght P, et al (2011) Genotypic and phenotypic characterization of the *Acinetobacter calcoaceticus- Acinetobacter baumannii* complex with the proposal of *Acinetobacter pittii* sp. nov. (formerly *Acinetobacter* genomic species 3) and *Acinetobacter nosocomialis* sp. nov. (formerly *Acinetobacter* genomic species 13TU). Res Microbiol 162: 393–404. 21320596 10.1016/j.resmic.2011.02.006

[3] Doughari H, Ndakidemi P, Human I, Benade S (2011) The ecology, biology and pathogenesis of *Acinetobacter* spp.: an overview. Microbes Environ 26: 101–112. 21502736 10.1264/jsme2.me10179

[4] Mussi MA, Limansky AS, Relling V, Ravasi P, Arakaki A, et al (2011) Horizontal gene transfer/assortative recombination within the *Acinetobacter baumannii* clinical population provides genetic diversity at the single *carO* gene encoding a major outer membrane protein channel. J Bacteriol 193: 4736–4748. 21764928 10.1128/JB.01533-10 PMC3165691

[5] Mussi MA, Gaddy JA, Cabruja M, Arivett BA, Viale AM, et al (2010) The opportunistic human pathogen *Acinetobacter baumannii* senses and responds to light. J Bacteriol 192: 6336–6345. 20889755 10.1128/JB.00917-10 PMC3008525

[6] Gaddy JA, Actis LA (2009) Regulation of *Acinetobacter baumannii* biofilm formation. Future Microbiol 4: 273–278. 19327114 10.2217/fmb.09.5 PMC2724675

[7] Gaddy JA, Tomaras AP, Actis LA (2009) The *Acinetobacter baumannii* 19606 OmpA protein plays a role in biofilm formation on abiotic surfaces and the interaction of this pathogen with eukaryotic cells. Infect Immun 77: 3150–3160. 19470746 10.1128/IAI.00096-09 PMC2715673

[8] Tomaras AP, Dorsey CW, Edelmann RE, Actis LA (2003) Attachment to and biofilm formation on abiotic surfaces by *Acinetobacter baumannii*: involvement of a novel chaperone-usher pili assembly system. Microbiol 149: 3473–3484. 10.1099/mic.0.26541-0 14663080

[9] Dijkshoorn L, van Harsselaar B, Tjernberg I, Bouvet PJM, Vaneechoutte M (1998) Evaluation of amplified ribosomal DNA restriction analysis for identification of *Acinetobacter* genomic species. System Appl Microbiol 21: 33–39. 9786720 10.1016/S0723-2020(98)80006-4

[10] Towner KJ, Bergogne-Berezin E, Fewson CA (1991) *Acinetobacter*: portrait of a genus. In K. J. Towner, E. Bergogne-Berezin, and C. A. Fewson, editors. The biology of *Acinetobacter*. New York: Plenum Press. 1–24.

[11] O’Toole GA, Pratt LA, Watnick PI, Newman DK, Weaver VB, et al (1999) Genetic approaches to the study of biofilms. Methods Enzymol 310: 91–109. 10547784 10.1016/s0076-6879(99)10008-9

[12] Sambrook J, Russell DW (2001) Molecular cloning: a laboratory manual, 3rd ed. Cold Spring Harbor: Cold Spring Harbor Laboratory Press.

[13] Hunger M, Schmucker R, Kishan V, Hillen W (1990) Analysis and nucleotide sequence of an origin of DNA replication in *Acinetobacter calcoaceticus* and its use for *Escherichia coli* shuttle plasmids. Gene 87: 45–51. 2185139 10.1016/0378-1119(90)90494-c

[14] Performance Standards for Antimicrobial Disk Susceptibility Tests: Approved Standard M2-A9. (2006) Pennsylvania: Clinical and Laboratory Standard Institute.

[15] Punta M, Coggill PC, Eberhardt RY, Mistry J, Tate J, et al (2012) The Pfam protein families database. Nucleic Acids Research Database Issue 40: D290–D301. 10.1093/nar/gkr1065 PMC3245129 22127870

[16] Thompson JD, Higgins DG, Gibson TJ (1994) CLUSTAL W: improving the sensitivity of progressive multiple sequence alignment through sequence weighting, position-specific gap penalties and weight matrix choice. Nucleic Acids Res 22: 4673–4680. 7984417 10.1093/nar/22.22.4673 PMC308517

[17] Gomelsky M, Klug G (2002) BLUF: a novel FAD-binding domain involved in sensory transduction in microorganisms. Trends Biochem Sci 27: 497–500. 12368079 10.1016/s0968-0004(02)02181-3

[18] Hall TA (1999) BioEdit: a user-friendly biological sequence alignment editor and analysis program for Windows 95/98/NT. Nucleic Acids Symp Ser 41: 95–98.

[19] Felsenstein J (1989) PHYLIP-phylogeny inference package (version 3.2). Cladistics 5: 164–166.

[20] Diancourt L, Passet V, Nemec A, Dijkshoorn L, Brisse S (2010) The population structure of *Acinetobacter baumannii*: expanding multiresistant clones from an ancestral susceptible genetic pool. PLoS ONE 5: e10034. 20383326 10.1371/journal.pone.0010034 PMC2850921

[21] Nemec A, Vaneechoutte M, Dijkshoorn L (2009) *Acinetobacter beijerinckii* sp. nov. and *Acinetobacter gyllenbergii* sp. nov., haemolytic organisms isolated from humans. Int J Syst Evol Microbiol 59: 118–124. 19126734 10.1099/ijs.0.001230-0

[22] Jung A, Domratcheva T, Tarutina M, Wu Q, Ko W, et al (2005) Structure of a bacterial BLUF photoreceptor: Insights into blue light-mediated signal transduction. Proc Nat Acad Sci USA 102: 12350–12355. 16107542 10.1073/pnas.0500722102 PMC1194903

[23] Zan J, Cicirelli EM, Mohamed NM, Sibhatu H, Kroll S, et al. (2012) A complex LuxR-LuxI type quorum sensing network in a roseobacterial marine sponge symbiont activates flagellar motility and inhibits biofilm formation.Mol Microbiol In press. 10.1111/j.1365-2958.2012.08149.x PMC3429658 22742196

[24] Pesavento C, Becker G, Sommerfeldt N, Possling A, Tschowri N, et al (2008) Inverse regulatory coordination of motility and curli-mediated adhesion in *Escherichia coli* . Genes Dev 22: 2434–2446. 18765794 10.1101/gad.475808 PMC2532929

[25] Caiazza NC, Merritt JH, Brothers KM, O'Toole GA (2007) Inverse regulation of biofilm formation and swarming motility by *Pseudomonas aeruginosa* PA14. J Bacteriol 189: 3603–3612. 17337585 10.1128/JB.01685-06 PMC1855903

[26] McQueary CN, Actis LA (2011) *Acinetobacter baumannii* biofilms: variations among strains and correlations with other cell properties. J Microbiol 49: 243–250. 21538245 10.1007/s12275-011-0343-7

[27] Bouvet PJM, Grimont P (1986) Taxonomy of the genus *Acinetobacter* with the recognition of *Acinetobacter baumannii* sp. nov., *Acinetobacter hemolyticus* sp. nov., *Acinetobacter johnsonii* sp. nov., and *Acinetobacter junii* sp. nov. and emended descriptions of *Acinetobacter calcoaceticus* and *Acinetobacter lwoffii* . Int J Syst Bacteriol 36: 228–240.

[28] Vaneechoutte M, Young DM, Ornston LN, De Baere T, Nemec A, et al (2006) Naturally transformable *Acinetobacter* sp. strain ADP1 belongs to the newly described species *Acinetobacter baylyi* . Appl Environ Microbiol 72: 932–936. 16391138 10.1128/AEM.72.1.932-936.2006 PMC1352221

[29] Nemec A, Musílek M, Sedo O, De Baere T, Maixnerová M, et al (2010) *Acinetobacter bereziniae* sp. nov. and *Acinetobacter guillouiae* sp. nov., to accommodate *Acinetobacter* genomic species 10 and 11, respectively. Int J Syst Evol Microbiol 60: 896–903. 19661501 10.1099/ijs.0.013656-0

[30] Carr EL, Kämpfer P, Patel BK, Gürtler V, Seviour RJ (2003) Seven novel species of *Acinetobacter* isolated from active sludge. Int J Syst Evol Microbiol 53: 953–63. 12892111 10.1099/ijs.0.02486-0

[31] Anandham R, Weon HY, Kim SJ, Kim YS, Kim BY, et al (2010) *Acinetobacter brisouii* sp. nov., isolated from a wetland in Korea. J. Microbiol. 48: 36–39. 10.1007/s12275-009-0132-8 20221727

[32] Zhan Y, Yan Y, Zhang W, Yu H, Chen M, et al (2011) Genome sequence of *Acinetobacter* calcoaceticus PHEA-2, isolated from industry wastewater. J Bacteriol 193: 2672–3. 21441526 10.1128/JB.00261-11 PMC3133148

[33] Malhotra J, Anand S, Jindal S, Raman R, Lal R (2012) *Acinetobacter indicus* sp. nov., isolated from hexachlorocyclohexane (HCH) dumpsite. Int J Syst Evol Microbiol In Press. 10.1099/ijs.0.037721-0 22247213

[34] Seifert H, Dijkshoorn L, Gerner-Smidt P, Pelzer N, Tjernberg I, et al (1997) Distribution of *Acinetobacter* species on human skin: comparison of phenotypic and genotypic identification methods. J Clin Microbiol 35: 2819–2825. 9350741 10.1128/jcm.35.11.2819-2825.1997 PMC230069

[35] Jung J, Baek JH, Park W (2010) Complete genome sequence of the diesel-degrading *Acinetobacter* sp. strain DR1. J Bacteriol 192: 4794–4795. 20639327 10.1128/JB.00722-10 PMC2937415

[36] Nemec A, Dijkshoorn L, Cleenwerck I, De Baere T, Janssens D, et al (2003) *Acinetobacter parvus* sp. nov., a small-colony forming species isolated from human clinical specimens. Int J Syst Evol Microbiol 53: 1563–1567. 13130049 10.1099/ijs.0.02631-0

[37] Nishimura Y, Kairiyama E, Shimadzu M, Iizuka H (1981) Characterization of a radiation-resistant *Acinetobacter* . Z Allg Mikrobiol 21: 125–30. 7269645 10.1002/jobm.3630210208

[38] Vaz-Moreira I, Novo A, Hantsis-Zacharov E, Lopes AR, Gomila M, et al (2011) *Acinetobacter rudis* sp. nov., isolated from raw milk and raw wastewater. Int J Syst Evol Microbiol 61: 2837–2843. 21239566 10.1099/ijs.0.027045-0

[39] Nemec A, de Baere T, Tjernberg I, Vaneechoutte M, van der Reijden TJ, et al (2001) *Acinetobacter ursingii* sp. nov. and *Acinetobacter schindleri* sp. nov., isolated from human clinical specimens. Int J Syst Evol Microbiol 51: 1891–1899. 11594623 10.1099/00207713-51-5-1891

[40] Vaneechoutte M, Nemec A, Musílek M, van der Reijden TJ, van den Barselaar M, et al (2009) Description of *Acinetobacter venetianus* ex Di Cello *et al.*, 1997 sp. nov.Int J Syst Evol Microbiol 59: 1376–1381. 19502319 10.1099/ijs.0.003541-0

[41] Soslau G, Russell JA, Spotila JR, Mathew AJ, Bagsiyao P, et al (2011) *Acinetobacter* sp. HM746599 isolated from leatherback turtle blood. FEMS Microbiol Lett 322: 166–171. 21707734 10.1111/j.1574-6968.2011.02346.x

